# Supplementary material for: Causal interplay between lactose intolerance and gut microbiota: a combined bidirectional Mendelian randomization and in vivo validation study
Source: Front Nutr. 2026 Jun 1;13:1803337. doi: 10.3389/fnut.2026.1803337 (PMC13265576; doi:10.3389/fnut.2026.1803337)
Supplement: Supplementary file 1 [file Data_Sheet_1.zip › supplementary materials/Forward/forest plot/ebi-a-GCST90027777.finngen_R12_E4_LACTONAS.pdf]

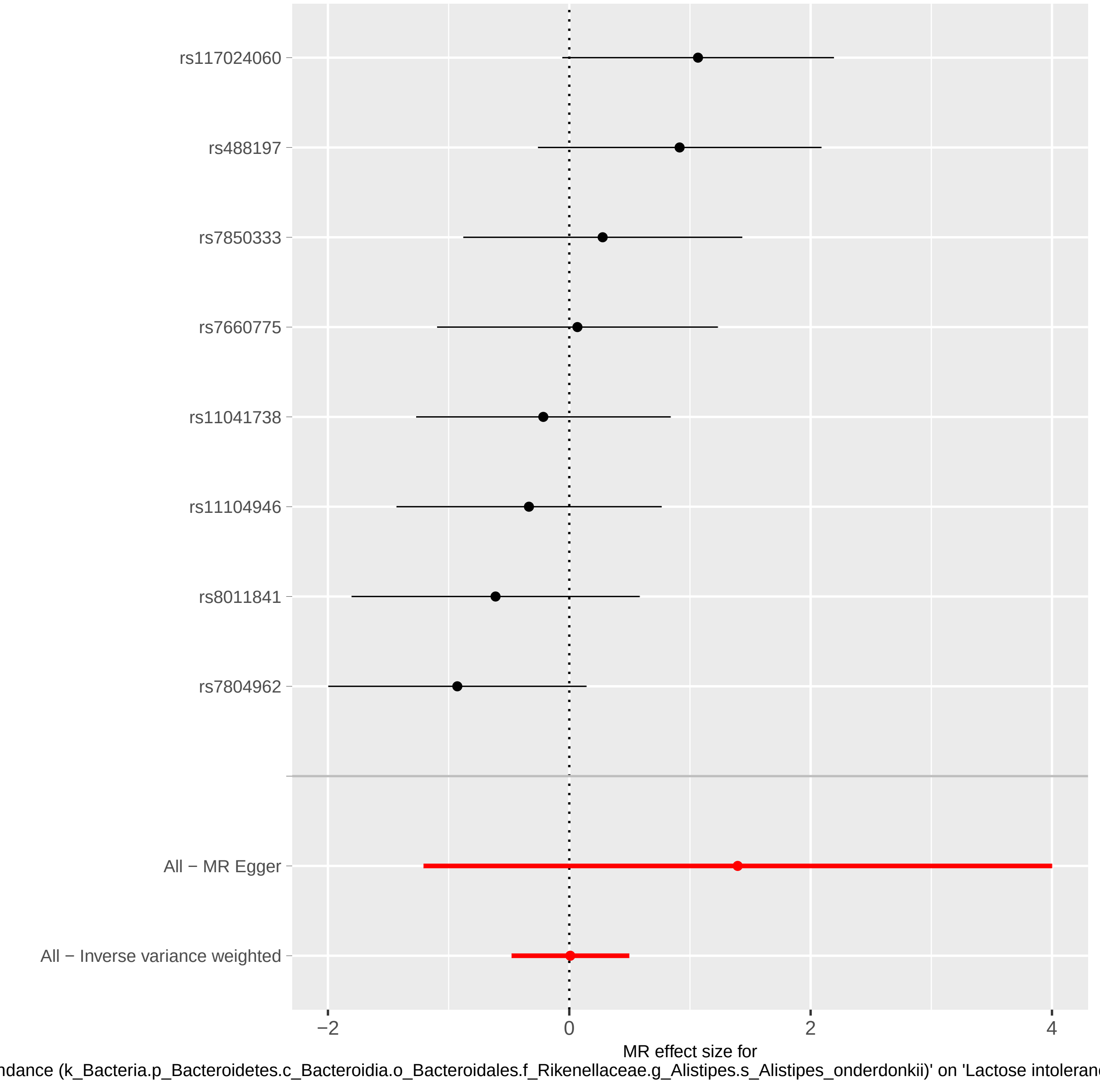

rs117024060
rs488197
rs7850333
rs7660775
rs11041738
rs11104946
rs8011841
rs7804962
All − MR Egger
All − Inverse variance weighted
−2
0
2
4
MR effect size for
ndance (k_Bacteria.p_Bacteroidetes.c_Bacteroidia.o_Bacteroidales.f_Rikenellaceae.g_Alistipes.s_Alistipes_onderdonkii)' on 'Lactose intoleran
